# The brain–gut–skin axis in inflammatory and disfiguring skin diseases: mechanistic insights, clinical correlations, and therapeutic strategies

**DOI:** 10.3389/fimmu.2026.1737303

**Published:** 2026-02-27

**Authors:** Zijian Guo, Jiao Yang, Rui Zang, Yixuan Yang, Qingnan Wang, Chenchen Xu

**Affiliations:** Department of Dermatology, Guanganmen Hospital, China Academy of Chinese Medical Sciences, Beijing, China

**Keywords:** brain–gut–skin axis, gut microbiota, inflammatory skin diseases, integrative medicine, neuroimmunology, psychodermatology

## Abstract

Emerging evidence suggests that the brain–gut–skin axis (BGSA) plays a critical role in the pathogenesis of inflammatory and disfiguring skin diseases. Conditions such as acne, atopic dermatitis, psoriasis, rosacea, vitiligo, and alopecia areata, once regarded as localized disorders driven mainly by cutaneous immune dysfunction, are now recognized as systemic conditions associated with neuroendocrine stress responses, gut microbial dysbiosis, and chronic low-grade inflammation. Mechanistic studies elucidate the intricate interorgan communication mediated by microbial metabolites (e.g., short-chain fatty acids and tryptophan derivatives), cytokine networks, neuropeptides, and hypothalamic–pituitary–adrenal (HPA) axis signaling. Building on these insights, therapeutic strategies are evolving rapidly. Microbiome-directed interventions (probiotics, postbiotics, dietary modification, and fecal microbiota transplantation), together with psychoneuroimmunological approaches, have shown potential to alleviate disease severity. Integrative therapies, including traditional herbal medicine, offer promising effects; however, we emphasize that mechanistic depth and robust clinical validation for these modalities are currently limited. This review integrates mechanistic findings, clinical correlations, and emerging therapeutic approaches, while critically distinguishing between correlation and causation. Future studies should emphasize longitudinal multi-omics analyses and standardized clinical trials to clarify causal pathways and guide precision, patient-centered management for systemic and cutaneous health.

## 1 Introduction

Recent advances in dermatology and immunology have reshaped the understanding of chronic inflammatory and disfiguring skin diseases. Conditions such as psoriasis, atopic dermatitis (AD), acne vulgaris, and rosacea, once regarded as isolated cutaneous disorders, are now recognized as manifestations of systemic dysregulation affecting multiple organ systems. Central to this new perspective is the brain–gut–skin axis (BGSA), a complex bidirectional communication network that integrates neuroendocrine, immune, and microbial signals, collectively influencing skin health and disease pathogenesis (1, 2).

To address the conceptual gaps in existing models, it is crucial to delineate how the BGSA differs from established linear axes. While the gut–brain and gut–skin axes have been extensively characterized as distinct pathways, the BGSA represents a higher-order, triangular regulatory network. Unlike the gut–skin axis, which primarily focuses on metabolic and immune interactions, or the gut–brain axis, which centers on neurobehavioral signaling, the BGSA posits that cutaneous homeostasis is maintained by a continuous tripartite dialogue. The distinct novelty and added value of this integrated concept lie in its ability to map non-linear feedback loops—specifically, how cutaneous inflammation can retrogradely influence the central nervous system (CNS) activity via the skin-brain axis (e.g., itch-anxiety cycles), creating a self-amplifying loop that linear models fail to capture. Recognizing this triangular connectivity—rather than viewing these systems in isolation—is critical for understanding why single-target therapies often fail in complex inflammatory dermatoses.

The concept of the BGSA was first introduced by Stokes and Pillsbury in the 1930s. Based on clinical observations and experimental findings, they proposed a connection between emotional stress, gut physiology, and skin conditions (3). In 2010, the BGSA was more precisely defined as a complex bidirectional communication network that integrates neural, endocrine, metabolic, immune, and microbial signaling pathways (4, 5). Subsequent studies demonstrated that gut-derived signals can modulate CNS function, while brain activity can influence gastrointestinal processes such as motility, sensation, and secretion (6). However, recent evidence warns against overemphasizing the HPA axis as the sole unifying driver across all diseases. As highlighted in recent reports on stress pathology (7), stress responses are highly heterogeneous and involve complex neurocircuitry (e.g., amygdala-prefrontal cortex loops) and neurochemical dysregulation (including orexin, glutamate, and neuroinflammation) that extend beyond simple HPA axis hyperactivation. Thus, the BGSA must be viewed as a flexible network where stress acts as a variable modifier—sometimes involving cortisol, but often driven by distinct neuro-immune or neurotransmitter pathways depending on the specific disease context.

Therapeutic implications of the BGSA are profound but require rigorous validation. Modulating gut microbiota through probiotics, prebiotics, diet, and even fecal microbiota transplantation (FMT) has shown promise in preclinical and early clinical studies for psoriasis and AD (8–12). Beyond the gut, recent investigations (13, 14) have underscored the importance of the skin microbiome, suggesting that direct cutaneous modulation can synergize with systemic interventions to improve skin disease outcomes. Psychodermatologic interventions that reduce stress can also mitigate neuroendocrine dysregulation and potentially improve skin outcomes (15, 16). Regarding Traditional Chinese Medicine (TCM) and herbal formulations, while preliminary findings are promising, mechanistic depth and clinical validation are currently limited. Caution is needed to distinguish between hypothesis-generating evidence from animal models and clinically actionable findings in humans.

This review provides a comprehensive synthesis of current knowledge on the BGSA, emphasizing molecular and cellular mechanisms linking the gut microbiome, neuroimmune communication, and skin inflammation. We aim to critically distinguish between correlation and causation, examining clinical associations in major inflammatory and disfiguring skin diseases, along with therapeutic approaches targeting this axis. A deeper understanding of the integrated BGSA network may enable the development of innovative diagnostic and precision therapeutic strategies that move beyond symptomatic management to address underlying systemic causes.

## 2 The BGSA: mechanistic insights

The BGSA functions as a dynamic bidirectional communication network linking the CNS, gastrointestinal tract, and skin through interconnected neuroendocrine, immune, and microbial pathways. Disturbance of this homeostatic balance can initiate or aggravate inflammatory and disfiguring skin disorders. Recent progress has clarified key molecular mediators and signaling pathways that govern the intricate interactions among these three organ systems.

### 2.1 Components of the axis

The BGSA consists of three interconnected systems: (1) the CNS, encompassing the hypothalamic–pituitary–adrenal (HPA) axis and autonomic pathways; (2) the gastrointestinal (GI) tract, including the intestinal microbiota and mucosal immune system; and (3) the skin, which serves both as a physical barrier and as an active neuroendocrine and immune organ.

Communication among these systems occurs bidirectionally through neuroendocrine signaling, microbial metabolites, immune mediators, and neural circuits such as the vagus nerve. The HPA axis plays a central role by releasing corticotropin-releasing hormone (CRH), adrenocorticotropic hormone (ACTH), and cortisol in response to stress, thereby influencing gut barrier integrity, altering microbial composition, and regulating skin inflammation.

### 2.2 Key mechanistic pathways of interaction

#### 2.2.1 Psychological stress: from HPA axis to multi-system dysregulation

Psychological stress is a well-established precipitating factor for skin diseases. Classically, this is attributed to the activation of the HPA axis and sympathetic nervous system, resulting in the systemic release of glucocorticoids (e.g., cortisol), catecholamines (e.g., epinephrine), and neuropeptides such as Substance P and calcitonin gene-related peptide (CGRP) (1, 16, 17).

Mechanistically, this cascade initiates in the hypothalamus. CRH secretion stimulates pituitary ACTH and adrenal cortisol production. Cortisol binds to cutaneous glucocorticoid receptors, paradoxically promoting additional local CRH release and proinflammatory cytokine expression in a feed-forward loop. Concurrently, stress alters peripheral nerve function, enhancing Substance P release to drive neurogenic inflammation (18). These mediators exert pleiotropic effects: in the gut, they increase permeability (“leaky gut”) and disrupt microbial composition (17, 19–22); in the skin, cortisol suppresses collagen synthesis and impairs barrier function (23), while catecholamines and neuropeptides trigger mast cell degranulation and vascular hyperreactivity (22, 24–27).

However, the “cortisol-centric” view is evolving. Recent neurobiological evidence indicates that the HPA axis is not the sole mediator of stress pathology. As highlighted in a 2025 review by Prajapati et al. (7), chronic stress triggers a heterogeneous “multi-system” dysregulation involving specific neurocircuitry abnormalities (e.g., amygdala hyperactivity) and neurochemical imbalances in the orexinergic, GABAergic, and glutamatergic systems. Crucially, chronic stress can lead to HPA axis “blunting” (hypocortisolism due to impaired feedback) rather than sustained hyperactivation. In such states, pathology is propagated via compensatory sympathetic hyperactivity and direct neuro-immune crosstalk. This distinction is vital for dermatology, as it explains how stress exacerbates skin disease via direct autonomic signaling even when systemic cortisol levels are not elevated.

#### 2.2.2 Gut microbial dysbiosis and metabolite signaling

The gut microbiota serves as a metabolic engine for the BGSA. A balanced microbiome generates essential immunoregulatory metabolites, particularly short-chain fatty acids (SCFAs like butyrate). SCFAs enhance regulatory T cell (Treg) differentiation and suppress Th17 activity, thereby preserving systemic immune homeostasis.

In contrast, disease-associated dysbiosis—often characterized by a loss of *Faecalibacterium prausnitzii* and reduced diversity—compromises this metabolic support (28, 29). The resulting decline in SCFA production, combined with elevated lipopolysaccharide (LPS) translocation, engages Toll-like receptor 4 (TLR4) to ignite systemic inflammation (21, 28). Furthermore, the gut acts as a neuroendocrine organ: microbes synthesize neuroactive metabolites (GABA, serotonin, dopamine) that modulate mood and stress responses, contributing to the psychiatric comorbidities frequently observed in dermatologic patients (30–33). Recent findings further highlight reciprocal gut–skin interactions mediated by microbial metabolites and signaling molecules, reinforcing the concept of skin inflammation as a systemic process (34).

Despite these insights, a significant challenge in interpreting BGSA research is the inconsistency of reported microbial signatures across studies. A striking example is the Firmicutes/Bacteroidetes (F/B) ratio in psoriasis patients, where results are conflicting: while Xiao et al. (35) reported a significant increase in the F/B ratio, Huang et al. (36) observed a significant decrease driven by an enrichment of Bacteroidetes (37). These discrepancies likely arise from methodological heterogeneity, including differences in DNA extraction protocols, sequencing platforms (16S rRNA vs. shotgun metagenomics), and bioinformatic pipelines (38, 39). Furthermore, patient-specific factors such as diet, geography, antibiotic history, and host genetics (e.g., HLA polymorphisms) act as confounders (40, 41). Therefore, identifying functional metabolic shifts (e.g., loss of SCFA production) may be more clinically relevant than cataloging taxonomic abundance changes alone.

#### 2.2.3 Immune system crosstalk and cytokine networks

Cytokines serve as the long-range messengers of the BGSA. Disruption of the gut barrier or CNS stress pathways induces a systemic surge in IL-1β, IL-6, IL-17, TNF-α, and IFN-γ (42, 43).

These cytokines exhibit trans-compartmental mobility: they can cross the blood–brain barrier (BBB), modulate CNS signaling and simultaneously act on skin-resident immune cells (44). The “leaky gut” hypothesis posits that bacterial components (e.g., LPS, DNA) entering circulation engage pattern recognition receptors such as TLRs on keratinocytes and dendritic cells, leading to the release of pro-inflammatory cytokines (e.g., IL-23, IL-17, TNF-α) (45). IL-17A, produced by Th17 cells and γδ T cells, represents a key inflammatory mediator common to both psoriasis and gastrointestinal disorders such as Crohn’s disease. The convergence of these cytokine pathways indicates that systemic immunomodulatory therapies may alleviate both gut and skin inflammation (46).

Skin inflammation elevates systemic cytokine levels, which modulate the HPA axis and intestinal immune activity, leading to microbial imbalance and increased barrier permeability. Immune cells activated in the gut can migrate to the skin, and skin-derived immune cells can return to the gut, with dysregulated immune cell trafficking recognized as a key mechanism linking inflammation across these organ systems (47, 48).

#### 2.2.4 Integrated systems perspective and therapeutic implications

In summary, the BGSA constitutes a complex network where neuroendocrine, immune, and microbial pathways intersect to regulate dermatologic health. Disruption of this system connects psychological stress, gut dysbiosis, and immune imbalance to the initiation and progression of inflammatory skin diseases. From a systems-level perspective, the BGSA offers a conceptual framework for holistic and multi-targeted therapeutic strategies. Dietary interventions incorporating polyphenol-rich foods, probiotics, and prebiotics can help restore microbial equilibrium, reinforce barrier integrity, and mitigate skin inflammation. Concurrently, psychological approaches such as mindfulness and cognitive-behavioral therapy can modulate stress-related neuroendocrine activity and attenuate systemic inflammation. While recognizing that correlation does not equal causation, accumulating evidence validates the functional significance of these pathways, advancing dermatology toward a more integrative understanding that extends beyond localized skin treatment (**Table 1**).

**Table 1:** Summary of mechanisms.

Mechanism	Key players	Evidence summary
Neuroendocrine stress signaling	HPA axis, cortisol, CGRP, Substance P	Stress-induced disruption of the gut barrier observed in patients with atopic dermatitis and psoriasis
Microbial metabolites	SCFAs, polyphenol derivatives, and bile acids	SCFAs promote regulatory T cell differentiation, while dysbiosis enhances inflammatory responses
Microbial translocation	LPS, bacterial DNA	Entry of microbial components into circulation activates TLR pathways in the skin, worsening inflammation
Neuroimmune modulation by metabolites	GABA, serotonin, dopamine	Gut-derived neuromodulators influence neurogenic and immune signaling in the skin
Immune cell trafficking	T cells, innate immune cells	Gut-primed immune cells migrate to the skin; cytokines from skin inflammation feed back to modulate gut immunity
Diet & probiotics as modulating tools	Polyphenols, probiotics	Dietary and probiotic interventions enhance barrier function and immune regulation through gut–skin communication

**Figure 1:**
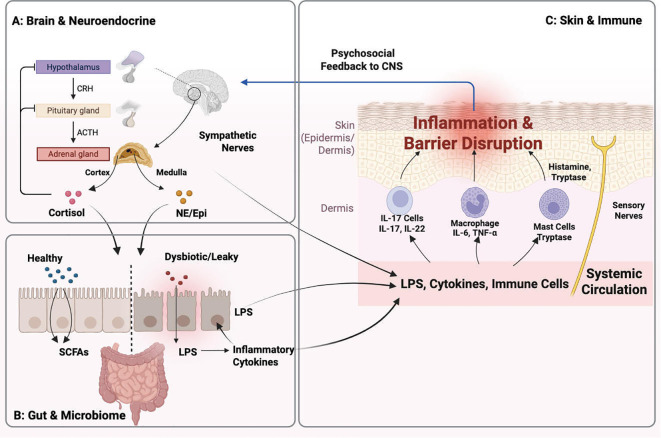
Molecular and Cellular Mechanisms of the Brain–Gut–Skin Axis.

**Figure 1**: The Integrated Mechanistic Network of the Brain–Gut–Skin Axis (BGSA). This schematic illustrates the continuous, triangular regulatory loops linking neuroendocrine, microbial, and immune systems. (A) Brain & Neuroendocrine Signaling (Top-Down): Psychological stress activates the hypothalamus and pituitary. The pituitary releases ACTH, stimulating the adrenal cortex to secrete Cortisol. Concurrently, sympathetic nerves stimulate the adrenal medulla to release Catecholamines (Epi, NE). These stress mediators target both the gut and the skin. (B) Gut & Microbiome (Bottom-Up): Stress mediators disrupt the gut barrier. A healthy gut (left) produces beneficial short-chain fatty acids (SCFAs, shown as blue spheres). A dysbiotic/leaky gut (right) allows bacterial lipopolysaccharide (LPS, red spheres) and inflammatory cytokines to translocate into the systemic circulation. (C) Skin & Immune Convergence: Systemic mediators (LPS, cytokines) and immune cells are delivered to the skin dermis via circulation. Specific immune cells are activated: Th17 Cells release IL-17 and IL-22; Macrophages release TNF-α and IL-6; and Mast Cells, activated by sensory nerves, release histamine and tryptase. These signals converge to cause epidermal inflammation and barrier disruption. The cycle is completed as chronic skin disease generates psychosocial distress, feeding back to the CNS (blue curved arrow). (Abbreviations: ACTH, Adrenocorticotropic Hormone; CNS, Central Nervous System; Epi, Epinephrine; NE, Norepinephrine; SCFAs, Short-Chain Fatty Acids.).

## 3 Clinical correlations: the BGSA in dermatological diseases

It is important to note that while the BGSA provides a unifying framework, the weight of its components varies by disease and individual context. In conditions like atopic dermatitis, stress often acts as a potent ‘modifier’ or ‘exacerbating factor’ that amplifies pre-existing barrier defects, rather than serving as the primary initiating driver. The following sections explore these nuances across specific dermatoses (**Table 2**).

**Table 2:** Brain–gut–skin axis (BGSA) in dermatological diseases.

Disease	Core pathophysiology	Microbial features	Neuroendocrine/Immune mechanisms	Intervention strategies
Acne Vulgaris	Chronic inflammatory disorder of pilosebaceous units; stress-induced HPA activation; mTOR signaling dysregulation	Gut dysbiosis: ↓*Actinobacteria*, ↑*Proteobacteria*, ↓diversity; *Cutibacterium*, *Acinetobacter*, *Roseomonas*	Stress → ↑CRH/cortisol → ↑IL-6/TNF-α; ↑Substance P → neurogenic inflammation	Probiotics, low-glycemic diet, psychological interventions (CBT), holistic management
Psoriasis	Chronic immune-mediated inflammation; HPA axis dysregulation; increased gut permeability	↓SCFA-producing bacteria (*Faecalibacterium prausnitzii*); ↑proinflammatory taxa (*Firmicutes*, *Proteobacteria*); SCFA/tryptophan metabolites modulate Treg/Th17	↑IL-17, IL-23, TNF-α; neurotransmitters (DA, 5-HT, GABA) modulate immunity	Probiotics, fecal microbiota transplantation (FMT), stress management, antioxidant-rich diets (Mediterranean/ketogenic), treat-to-target (T2T)
Atopic Dermatitis (AD)	Chronic relapsing inflammation; barrier dysfunction; HPA activation worsens inflammation	Skin: *S. aureus* dominance; Gut: ↓SCFA-producers, ↑Clostridium/*E. coli*; SCFA/tryptophan metabolites regulate immunity & neural signaling	Stress → ↑Th2 → ↑IL-4/IL-5/IL-13; LPS leakage → TLR2/4 activation	Oral probiotics (*Lactobacillus*, *Bifidobacterium*), topical commensals, GOS dietary interventions, microbiota transplantation, and neuromodulation
Rosacea	Chronic central facial inflammation; immune–neurovascular dysregulation	Gut dysbiosis: ↑*Lachnospiraceae*, ↑*Veillonellaceae*, ↓*Prevotellaceae*, ↓*Bifidobacteriaceae*; Demodex-associated microbial shifts	Neuropeptides (Substance P, CGRP, VIP) → vasodilation, mast cell degranulation, inflammation; HPA activation by stress	Standard therapies (metronidazole, azelaic acid, doxycycline), probiotics/gut microbiota modulation, topical probiotic formulations, stress management (mindfulness, CBT, sleep)
Hair Disorders (Alopecia, Telogen Effluvium)	Follicular biology dysfunction; stress disrupts follicular immune privilege	Gut dysbiosis; altered microbial metabolites	Stress → HPA axis → ↑CRH/Substance P → local neuroinflammation; Th1/Th17 skewing → ↑IFN-γ/IL-17	Probiotics, dietary modulation, barrier protection, combined neuroimmune interventions
Vitiligo	Immune-mediated melanocyte loss; stress & gut dysbiosis	Gut dysbiosis → endotoxin/LPS translocation	Stress → HPA & sympathetic activation → ↑IL-6/TNF-α/IFN-γ; oxidative stress and innate immunity dysregulation	Probiotics, dietary interventions, psychological support/stress management, integrated systems-based therapy

### 3.1 Acne vulgaris

Acne vulgaris is a chronic inflammatory disorder of the pilosebaceous unit that is increasingly recognized as a multifactorial disease involving the BGSA. Its pathogenesis can be structured into three converging pathways: stress-induced neuroendocrine activation, diet-microbiome-mediated metabolic dysregulation, and neurogenic inflammation.

First, psychoneuroendocrine factors initiate the cascade. Stress activates the HPA axis, resulting in elevated levels of cortisol and CRH. These hormones directly enhance sebaceous gland activity and stimulate the release of pro-inflammatory cytokines such as IL-6 and TNF-α. Crucially, recent findings indicate that local neurogenic inflammation acts synergistically with systemic stress. Elevated concentrations of Substance P have been detected in patients with acne, which amplifies inflammation by stimulating mast cell degranulation and increasing cutaneous innervation density. This creates a feed-forward loop where emotional stress exacerbates local neurogenic signals within the pilosebaceous unit (49, 50).

Second, the gut–skin metabolic interface plays a pivotal regulatory role. Diet serves as a critical regulator, where high-glycemic-load foods and dairy products activate the insulin and insulin-like growth factor-1 (IGF-1) signaling pathway. This pathway is central to the BGSA in acne because IGF-1 not only enhances lipogenesis but also interacts with the mammalian target of rapamycin complex 1 (mTORC1). Gut-derived metabolites reciprocally modulate this mTOR signaling, linking nutrient sensing directly to sebaceous gland function (51). Concurrently, dietary patterns shape the gut microbiome. Gut dysbiosis, characterized by reduced Actinobacteria, increased Proteobacteria, and diminished diversity, has been consistently documented in acne patients (52, 53). Such imbalance facilitates LPS translocation and reduces SCFA production, driving systemic inflammation (19, 54). Specific gastrointestinal associations further validate this link; for instance, a positive correlation exists between *Helicobacter pylori* infection and acne severity, potentially mediated by bacterial lipase activity and oxidative stress (55).

Finally, these systemic triggers converge to alter the skin microenvironment. Negative emotional states have been shown to correlate with specific microbial shifts (e.g., changes in *Cutibacterium* and *Acinetobacter*), suggesting that stress and dysbiosis are not isolated events but act synergistically (56). Elevated serum zonulin levels in acne patients further implicate gut barrier dysfunction (“leaky gut”) as the conduit allowing systemic inflammatory mediators to reach the skin (57). Therapeutic evidence reinforces this integrated model, as probiotic supplementation and low-glycemic diets alleviate severity likely by simultaneously dampening IGF-1 signaling and restoring microbial equilibrium (58, 59). Thus, acne represents a prototypical BGSA disorder where neuroendocrine stress and metabolic dysbiosis converge on the pilosebaceous unit.

### 3.2 Psoriasis

Psoriasis is a chronic immune-mediated skin disorder characterized by systemic inflammation and significant psychosocial comorbidities (60, 61). The BGSA drives psoriatic inflammation through a “triangular” interaction involving neuroendocrine dysregulation, gut barrier compromise, and specific metabolite-immune axes (47, 62).

The neuroendocrine arm of the axis is bidirectional. Patients frequently experience psychological distress, which activates the HPA axis and sympathetic nervous system. Beyond cortisol-mediated immunosuppression, gut microbes produce neurotransmitters—including dopamine, serotonin, and γ-aminobutyric acid (GABA)—that regulate neuroimmune pathways. Dopamine, for instance, has been shown to influence T-cell migration and promote keratinocyte proliferation (63). Conversely, proinflammatory cytokines (IL-1β, TNF-α, IL-6) released during psoriatic flares can cross the blood–brain barrier, modulating neuronal and glial activity, potentially driving the high prevalence of depression in these patients (48, 64).

The gut–skin connection in psoriasis is marked by profound dysbiosis and barrier loss. Recent research reveals reduced microbial richness, depletion of SCFA-producing bacteria (e.g., *Faecalibacterium prausnitzii*), and enrichment of proinflammatory taxa (Firmicutes, Proteobacteria) (9). This dysbiosis functionally impairs the intestinal barrier, evidenced by elevated intestinal fatty acid binding protein (FABP) levels (65). Disruption of the mucus layer facilitates the translocation of bacterial DNA and endotoxins (LPS) into the circulation, triggering a systemic inflammatory response that primes cutaneous immunity (10, 66, 67). Mechanistic proof is provided by animal studies where fecal microbiota transplantation (FMT) from psoriasis patients successfully induces cutaneous inflammation in germ-free mice, whereas interventions like *Parabacteroides goldsteinii*–derived extracellular vesicles ameliorate it (68, 69).

The most critical mechanistic insight lies in the metabolite-immune convergence, specifically involving the Th17 axis. Gut microbiota metabolites significantly influence host immunity through aryl hydrocarbon receptor (AhR) signaling and histone acetylation. While SCFAs like butyrate promote regulatory T (Treg) cell differentiation (70, 71), tryptophan metabolism shows complex duality: indole-3-lactic acid (ILA) is protective, whereas indoxyl sulfate exacerbates inflammation via Th17 activation (72, 73). Furthermore, Innate Lymphoid Cells Group 3 (ILC3s), which reside in both gut and skin, act as key effectors. Expression of CD200R1 on ILC3s is essential for IL-23–induced STAT3 activation and optimal IL-17 secretion (74), underscoring the complex interplay between microbiota and immune receptors. Microbial metabolites differentially modulate ILC3 function; for example, acetate enhances while butyrate suppresses IL-22 production (75). This gut-tuned ILC3 activity, along with the systemic release of IL-23 and IL-17, completes the pathological loop linking gut dysbiosis to psoriatic plaques. Consequently, therapeutic strategies targeting this axis, from probiotics to stress management, aim to break this self-reinforcing cycle of neuro-metabolic inflammations (76–79).

### 3.3 Atopic dermatitis

Atopic dermatitis (AD) is a chronic relapsing inflammatory skin disorder characterized by severe pruritus, epidermal barrier dysfunction, and pronounced microbial dysbiosis. Within the BGSA framework, AD pathogenesis is driven by a unique “vicious cycle” involving dual-site microbial dysbiosis, neuroendocrine stress responses, and metabolic-immune convergence.

Significant alterations in both the skin and gut microbiomes have been documented in AD patients. First, AD is distinguished by simultaneous dysbiosis at two barrier sites: the skin and the gut. Cutaneous dysbiosis is typified by a dramatic loss of diversity and the dominance of *Staphylococcus aureus* (80). Recent mechanistic insights reveal that *S. aureus* is not merely a colonizer but a direct driver of neurogenic inflammation. It forms biofilms and releases phenol-soluble modulin α (PSMα), a virulence factor that intensifies barrier damage and, crucially, activates protease-activated receptor-2 (PAR-2) on sensory neurons. This direct microbe-nerve signaling induces non-histaminergic itch, perpetuating the scratch-itch cycle (81). Concurrently, gut dysbiosis presents as a depletion of SCFA-producing bacteria such as *Faecalibacterium prausnitzii* and *Bifidobacterium*, accompanied by overgrowth of proinflammatory species including *Clostridium difficile* and *Escherichia coli*. This systemic imbalance compromises intestinal epithelial integrity and facilitates the translocation of microbial antigens into circulation (82, 83).

Second, the gut-brain axis regulates cutaneous immunity through specific metabolite signaling. The bidirectional communication is mediated by microbial metabolites, including SCFAs, neurotransmitters, and tryptophan derivatives. SCFAs, particularly butyrate, exert epigenetic control to promote regulatory T-cell (Treg) differentiation and reinforce epithelial barrier integrity. Tryptophan metabolism represents a critical checkpoint in AD: while some indole derivatives exert anti-inflammatory effects through aryl hydrocarbon receptor (AhR) activation, dysregulated metabolism can generate proinflammatory signals via neurogenic pathways (82, 83). Furthermore, gut microbes modulate systemic cytokine production, including IL-10 and IFN-γ, which reciprocally influence neural signaling and stress responses (84).

Third, psychoneuroendocrine factors act as powerful aggravators of this unstable system. Psychological stress activates the HPA axis, resulting in the release of cortisol and CRH. These mediators shift the immune profile towards a Th2-type response (IL-4, IL-5, IL-13), which directly impairs epidermal barrier function and inhibits antimicrobial peptide production. Stress also increases intestinal permeability, facilitating LPS translocation that stimulates innate immune pathways including TLR2 and TLR4. This establishes a self-perpetuating BGSA loop: stress amplifies inflammation and barrier loss, leading to intensified pruritus, which in turn feeds back to aggravate psychological distress (85, 86).

Finally, therapeutic interventions validate this integrated multi-axis model. Microbiome-targeted strategies are evolving from general to specific modulation. Oral probiotics (*Lactobacillus* and *Bifidobacterium*) have been shown to restore the Th1/Treg balance and suppress proinflammatory signaling, improving SCORAD scores (87, 88). More precisely, topical “bacteriotherapy” using commensals like *Staphylococcus hominis* or *Staphylococcus epidermidis* successfully inhibits *S. aureus* colonization and decreases PSMα production, directly breaking the neurogenic itch cycle (89). Metabolic interventions, such as dietary galacto-oligosaccharides (GOS), modulate the axis by enriching beneficial microbiota, elevating SCFA levels, and normalizing brain neurotransmitters (90, 91). Emerging approaches like prebiotics, postbiotics, and microbiota transplantation further aim to restore microbial homeostasis and mitigate dysregulation of the gut–brain–skin axis in AD (92).

### 3.4 Rosacea

Rosacea is a chronic inflammatory dermatosis that predominantly affects the central face, presenting with persistent erythema, telangiectasia, papules, pustules, and, in some cases, phymatous or ocular manifestations. Viewed through the lens of the BGSA, its pathogenesis is driven by a distinct neurovascular-microbial circuit, where central neuroendocrine dysregulation and gut metabolic shifts converge on a compromised cutaneous barrier.

First, the neurovascular arm constitutes the primary driver of the “flushing” phenotype. Clinically, neurogenic rosacea is defined by heightened flushing and erythema triggered by skin barrier dysfunction, thermal stimuli, stress, and hormonal fluctuations (93). The biological basis for this lies in central and peripheral neural dysregulation. Functional neuroimaging has revealed altered cerebral and limbic activity in rosacea patients, providing direct evidence of CNS involvement (94). Moreover, shared neuroendocrine pathways between the skin and gut can affect brain function through the autonomic nervous system and the HPA axis (95). This central signaling propagates to the skin via the dysregulated release of neuropeptides such as substance P, CGRP, and vasoactive intestinal peptide (VIP), which promotes vasodilation, mast cell degranulation, and immune activation (96, 97). Crucially, this system forms a bidirectional loop: brain-derived neurotrophic factor (BDNF) and serotonergic signaling link mood dysregulation to skin inflammation (98), while visible facial symptoms heighten psychosocial distress (depression, anxiety), reinforcing the pathological cycle (99).

Second, the gut–skin connection acts as a metabolic amplifier of this inflammation. Epidemiologically, this is supported by the high prevalence of gastrointestinal comorbidities such as *H. pylori* infection, small intestinal bacterial overgrowth (SIBO), and inflammatory bowel disease (IBD) (100). Microbiologically, next-generation sequencing reveals a specific “rosacea-associated dysbiosis.” While specific signatures vary—Nam et al. (101) reported increased *Acidaminococcus* and *Megasphaera*, Chen et al. (102) identified elevated *Bacteroides* and *Fusobacterium*, and Moreno-Arrones et al. (4) observed increased *Akkermansia muciniphila*—the functional consequence is consistent. Metabolomic profiling revealed 56 altered serum metabolites compared with healthy controls; notably, levels of 3, 4-dihydroxyphenylacetic acid were strongly correlated with *Bifidobacterium* and *Lactobacillus* abundance. This suggests that gut microbial imbalances do not act in isolation but actively modify the systemic metabolic landscape to drive pathophysiology (103).

Third, these systemic triggers alter the cutaneous microenvironment, leading to secondary microbial dysregulation. Microbial imbalance extends to the skin, centering on *Demodex* mites. Crucially, it is not just the mite burden but their bacterial cargo that drives heterogeneity. Murillo et al. found that mites from papulopustular rosacea carried higher proportions of Proteobacteria and Firmicutes, while those from erythematotelangiectatic rosacea were dominated by Actinobacteria. Pathogenic genera such as *Bartonella* and *Escherichia* were detected exclusively in mites from rosacea patients (104). These findings support the concept that the skin acts as a convergence point where neurovascular signals and gut-derived metabolites alter the terrain, facilitating complex host–microbe interactions that dictate the specific clinical phenotype (105).

Finally, therapeutic strategies targeting the BGSA validate this multi-nodal network. Conventional treatments (metronidazole, doxycycline) are now being complemented by axis-targeted interventions. Probiotics aim to restore regulation by modulating immune responses, suppressing neurogenic inflammation, and reducing vasodilation and TNF-α release (106, 107). Topically, formulations containing probiotic-derived components (e.g., *Vitreoscilla filiformis*) have been shown to reduce erythema and *Demodex* density (108). Simultaneously, stress management strategies, including mindfulness, cognitive behavioral therapy, and adequate sleep, have proven effective in interrupting the brain–skin feedback loop and improving overall disease control (109–111).

### 3.5 Hair disorders

Emerging evidence indicates that hair follicle biology and disorders such as alopecia areata (AA) and stress-related telogen effluvium can be understood within the integrated BGSA framework. Specifically, the hair follicle functions as a neuroimmune-sensitive “mini-organ” whose cycling is regulated by the tripartite integration of neuroendocrine signaling, microbial ecology, and immune privilege maintenance.

First, the neuroendocrine arm initiates the pathological cascade through stress signaling. Arck and colleagues first proposed that gut microbiota influence neurogenic skin inflammation and hair growth, demonstrating in mouse models that the ingestion of specific *Lactobacillus* strains attenuated stress-induced damage. Their findings supported a unified gut–brain–skin axis extending to hair follicle cycling and highlighted the role of neurohormonal mediators, including HPA-axis activation, sympathetic signaling, and neuropeptide release, as key links between stress, gut composition, and follicular responses (5). Mechanistically, chronic psychological stress elevates neuropeptides such as CRH and substance P in both the circulation and the perifollicular environment. Since hair follicles express functional CRH receptors (CRHR1 and CRHR2), exposure to these stress mediators directly inhibits hair growth and induces local neuroinflammation, indicating an intrinsic neuroendocrine feedback mechanism within the follicle (112).

Second, gut dysbiosis acts as a systemic modifier of this sensitivity. Gao et al. (113) reported that microbial dysbiosis impairs immune regulation, thereby increasing the host’s vulnerability to psychological stress-induced hair cycle disruption. Further expanding this concept, Feng (114) summarized potential mechanisms involving metabolic and barrier defects: the effects of microbial metabolites such as SCFAs and tryptophan derivatives, combined with the translocation of microbial components due to increased intestinal permeability, create a pro-inflammatory cytokine milieu that negatively affects follicular epithelial cells.

Third, these signals converge to cause the collapse of Follicular Immune Privilege (IP), the central pathogenic event in AA. Stress-induced neuroinflammation and gut-derived systemic signals synergize to disrupt the delicate immune homeostasis of the hair follicle. This collapse is characterized by the infiltration of CD8^+^ T cells and natural killer (NK) cells into the hair bulb, responding to increased expression of MHC I molecules, NKG2D ligands, and danger-associated signals on follicular keratinocytes. Concurrently, this systemic dysregulation promotes Th1/Th17 polarization, resulting in elevated IFN-γ and IL-17 levels that intensify local inflammatory cascades. Thus, the BGSA framework explains how psychological stress triggers autoreactive immune attacks and disturbs hair follicle cycling (112).

Finally, clinical interventions validate the therapeutic potential of this axis. Targeting the gut-metabolic interface has yielded measurable benefits. In a 12-week study, participants with hair loss and a high risk of metabolic syndrome received twice-daily supplementation with a specific probiotic formula. The treatment restored gut microbial balance, promoted hair growth, and reduced stress-related psychological and physiological responses. These results suggest that probiotics can simultaneously modulate microbial and neuroendocrine mechanisms underlying AA (115).

Collectively, these findings outline a coherent framework where targeted probiotics, dietary modulation, and barrier-protective approaches merit systematic investigation as dual-benefit strategies for hair loss.

### 3.6 Vitiligo

Vitiligo is a chronic immune-mediated depigmenting disorder characterized by the selective loss of functional melanocytes. The BGSA provides an integrative framework to explain how psychological distress and systemic metabolic shifts synergistically trigger cutaneous autoimmunity and oxidative stress.

First, the neuroendocrine arm initiates the systemic inflammatory cascade through psychological triggers. Chronic stress activates the HPA axis and sympathetic nervous system, modulating the systemic cytokine milieu. Circulating cytokines, including IL-6, TNF-α, and notably IFN-γ, can reach the skin and promote direct melanocyte injury (116, 117). This relationship is inherently bidirectional: visible depigmentation heightens psychosocial distress, which in turn exacerbates HPA axis activation, establishing a self-reinforcing pathological loop. Therefore, the disruption of this axis represents a key mechanism linking psychological stress to immune-mediated melanocyte loss.

Second, gut dysbiosis and altered microbial metabolism serve as systemic amplifiers of the disease. From the perspective of the BGSA, microbial imbalances account for the frequent comorbidity between vitiligo and psychiatric disorders. Gut dysbiosis facilitates the translocation of bacterial endotoxins such as LPS into circulation, leading to systemic inflammation. Crucially, tryptophan metabolism is a central metabolic checkpoint in this axis. Microbial metabolites of neurotransmitters contribute to emotional disturbances, while altered tryptophan metabolism further aggravates psychological dysfunction and modulates systemic immune responses (118). This metabolic dysregulation creates a pro-inflammatory environment that increases the host’s vulnerability to oxidative stress.

Third, these systemic neuroendocrine and microbial signals converge on the skin to drive innate and adaptive immune dysregulation. The convergence of stress-induced IFN-γ and gut-derived inflammatory mediators promotes the recruitment of autoreactive CD8+ T cells to the skin. Oxidative stress, which is central to vitiligo pathogenesis, is further influenced by these intersystemic interactions. The synergy between systemic cytokines and local oxidative stress leads to the collapse of melanocyte homeostasis and subsequent depigmentation (119, 120). Although the molecular details remain under investigation, this systems-based approach highlights the role of the BGSA in connecting emotional states and gut health to localized melanocyte destruction (116, 121).

Finally, therapeutic strategies targeting multiple nodes of the BGSA offer promising clinical outcomes. A multidisciplinary, patient-centered approach is essential for comprehensive care. Modulating the gut microbiota through probiotics, prebiotics, or dietary interventions has shown preliminary benefits in restoring microbial balance and reducing systemic inflammation (52, 122, 123). Simultaneously, psychological support and stress management (e.g., CBT) represent essential components that address the neuroendocrine drivers of the disease (119, 120, 124). Collectively, these interventions underscore the potential of a holistic strategy that not only targets depigmentation but also restores physiological and psychological balance across the interconnected brain–gut–skin network.

## 4 Therapeutic implications

Building on the mechanistic and clinical evidence across acne, psoriasis, AD, and rosacea, therapies targeting the BGSA have emerged as a promising integrative approach. The translational value of this framework lies in its ability to concurrently modulate neuroendocrine signaling, microbial metabolism, and immune homeostasis by intervening along the shared stress–HPA–gut–immune–skin pathway.

The evolution of microbiome-based interventions marks a significant shift from non-specific probiotic supplementation toward precision modulation strategies. While FMT effectively targets the dysbiotic gut ecosystem, direct manipulation of the cutaneous microbiome is gaining traction through the use of “postbiotics”—non-viable microbial cells or their bioactive metabolites. Prajapati et al. (13) recently highlighted this approach, noting that postbiotics (e.g., metabolites from *S. epidermidis*) can selectively inhibit pathogens like *S. aureus* in AD and acne, thereby restoring skin homeostasis without the safety risks associated with live bacterial proliferation. This precision-based scope has further expanded to include structural skin aging. Challa et al. (14) proposed the “Microbiome-Aging-Wrinkles Axis, “ demonstrating that oral or topical administration of *Lactobacillus* plantarum can downregulate Matrix Metalloproteinases (MMPs) and reduce oxidative stress, effectively preserving collagen integrity and positioning microbiome stability as a critical factor in delaying structural aging.

Complementing these microbial strategies, psychoneuroimmunological interventions aim to correct the “top-down” dysregulation induced by chronic stress. Mindfulness, cognitive-behavioral therapy (CBT), and other mind–body practices function as mechanistically active therapies rather than mere supportive care. By normalizing HPA axis activity and reducing systemic proinflammatory cytokines, these approaches address both the physiological drivers of dermatologic disease and the psychosocial burden shared among chronic conditions. The integration of these “brain-targeted” therapies ensures that the neuroendocrine triggers of the BGSA are addressed alongside peripheral symptoms.

Expanding the therapeutic repertoire further, integrative therapies such as traditional herbal medicines offer hypothesized multi-targeted modulation of these interconnected pathways. Botanical formulations are thought to act synergistically on microbial, immune, and neuroendocrine nodes, potentially restoring systemic balance where single-agent therapies may fail (125–127). However, a rigorous distinction must be maintained between mechanistic potential and clinical validation. While promising in animal models, many herbal interventions currently lack the support of robust, large-scale randomized controlled trials (RCTs). Future efforts must prioritize high-quality clinical evidence to elevate these multi-component modalities into standardized treatment protocols within the BGSA framework.

In conclusion, the therapeutic potential of the stress–HPA–gut–immune–skin axis necessitates a shift toward standardized and personalized medicine. By addressing a shared mechanistic pathway, interventions may exert therapeutic effects across multiple skin disorders. Nevertheless, challenges such as heterogeneous study designs and limited mechanistic clarity remain. Incorporating microbiome profiling, immune biomarkers, and psychological assessments into future clinical studies will be essential to fully harness the translational benefits of the BGSA and deliver holistic, patient-centered care.

## 5 Limitations

Despite expanding mechanistic and clinical evidence supporting the BGSA in inflammatory and disfiguring skin diseases, several limitations continue to constrain understanding and translation. Most clinical studies involve small cohorts, heterogeneous populations, and brief follow-up periods, which limit generalizability. Mechanistic insights are often based on preclinical or cross-sectional human data, making causal inference uncertain. Inconsistent methodologies for microbiome analysis, dietary interventions, and probiotic formulation further complicate data interpretation and cross-study comparison. Psychosocial and neuroendocrine variables, though increasingly recognized, remain inconsistently assessed, restricting comprehensive evaluation of the stress–HPA–gut–immune–skin pathway. Integrative therapies, including traditional herbal medicine, also lack rigorous mechanistic and clinical validation, with most evidence remaining preliminary.

Overcoming these challenges will require standardized, longitudinal multi-omics studies alongside well-designed clinical trials that integrate microbiome profiling, immune and neuroendocrine biomarkers, and psychosocial metrics. Only through such interdisciplinary research can causal mechanisms within the BGSA be clarified and personalized, and mechanism-based interventions effectively developed. **Table 3**.

**Table 3:** Key neuroendocrine and immune mediators in the brain-gut-skin axis.

Mediator	Source	Effects on gut	Effects on skin
Cortisol	HPA axis	Increases intestinal permeability	Suppresses cutaneous immunity
Substance P	Nerve endings	Increases gut motility and inflammation	Promotes neurogenic inflammation
Short-chain fatty acids	Gut microbiota	Maintain epithelial barrier integrity and exert anti-inflammatory effects	Exhibit anti-inflammatory activity and regulate keratinocyte differentiation
TNF-α	Immune cells	Increases intestinal permeability and promotes inflammation	Drives epidermal hyperplasia and inflammatory responses
IL-6	Immune and epithelial cells	Regulates mucosal immunity	Promotes inflammation and fibroblast proliferation

## 6 Conclusion

The BGSA provides a unified framework connecting psychological stress, gut microbiota imbalance, immune dysregulation, and cutaneous inflammation across chronic and disfiguring skin diseases such as acne, psoriasis, AD, and rosacea. Mechanistic research underscores the central involvement of this axis, yet it is crucial to recognize that stress and dysbiosis often act as “modifiers” rather than sole “drivers, “ varying in impact depending on the individual disease context.

Therapeutic strategies targeting the BGSA, including microbiome modulation, psychoneuroimmunological interventions, dietary optimization, and integrative therapies, offer promising avenues for holistic, multi-targeted management. However, current evidence remains constrained by heterogeneous study designs, limited sample sizes, and insufficient mechanistic validation.

Future research should emphasize longitudinal, multi-omics, and interdisciplinary studies to clarify causal relationships, identify predictive biomarkers, and design personalized treatment strategies. Bridging mechanistic insight with clinical translation may shift dermatologic care from symptom control toward precision, integrative, and patient-centered paradigms that improve both skin health and overall quality of life.
